# Pacemaker syndrome with sub-acute left ventricular systolic dysfunction in a patient with a dual-chamber pacemaker: consequence of lead switch at the header

**DOI:** 10.5830/CVJA-2016-081

**Published:** 2017

**Authors:** Mohammad Reeaze Khurwolah, Brian Zwelethini Vezi

**Affiliations:** Inkosi Albert Luthuli Central Hospital, Durban, KwaZulu-Natal, South Africa; Inkosi Albert Luthuli Central Hospital, Durban, KwaZulu-Natal, South Africa

**Keywords:** permanent pacemaker, lead switch, pacemaker syndrome, right ventricular-only pacing-induced left ventricular systolic dysfunction

## Abstract

In the daily practice of pacemaker insertion, the occurrence of atrial and ventricular lead switch at the pacemaker box header is a rare and unintentional phenomenon, with less than five cases reported in the literature. The lead switch may have dire consequences, depending on the indication for the pacemaker. One of these consequences is pacemaker syndrome, in which the normal sequence of atrial and ventricular activation is impaired, leading to sub-optimal ventricular filling and cardiac output. It is important for the attending physician to recognise any worsening of symptoms in a patient who has recently had a permanent pacemaker inserted. In the case of a dual-chamber pacemaker, switching of the atrial and ventricular leads at the pacemaker box header should be strongly suspected. We present an unusual case of pacemaker syndrome and right ventricular-only pacinginduced left ventricular systolic dysfunction in a patient with a dual-chamber pacemaker.

## Introduction

In the daily practice of pacemaker insertion, the occurrence of atrial and ventricular lead switch at the pacemaker box header is a rare and unintentional phenomenon, with less than five cases reported in the literature.^1^ The diagnosis of lead switch at the header is usually straightforward and is noticed quite early. If not, the possibility of this important complication should be considered in any patient presenting with ill-defined symptoms during pacemaker follow up.

Patients may present with a variety of symptoms, depending on the underlying rhythm, pacing rate and percentage of the paced beats. The symptoms of pacemaker syndrome are usually non-specific but often include dizzy spells, shortness of breath, fatigue, near-syncope, syncope or frank heart failure. The occurrence of right ventricular-only pacing-induced left ventricular systolic dysfunction has been well documented.^2^-^4^

## Case Report

A 40-year-old man presented with symptoms of undue fatigue and shortness of breath with minimal exertion. His resting heart rate was noted to reach 28 beats per minute (bpm) during waking hours. He was diagnosed with sick sinus syndrome and had a dual-chamber permanent pacemaker inserted. Subsequently, he reported feeling more ill and complained of dizziness, nearsyncope and syncope, worsening of shortness of breath, more fatigue, and what he described as a ‘strange heartbeat with fluttering’. Prior to the dual-chamber pacemaker insertion, he had undergone coronary angiography and left ventriculography, which showed normal epicardial coronary arteries and a left ventricular ejection fraction (LVEF) of 74%.

**Fig. 1.**
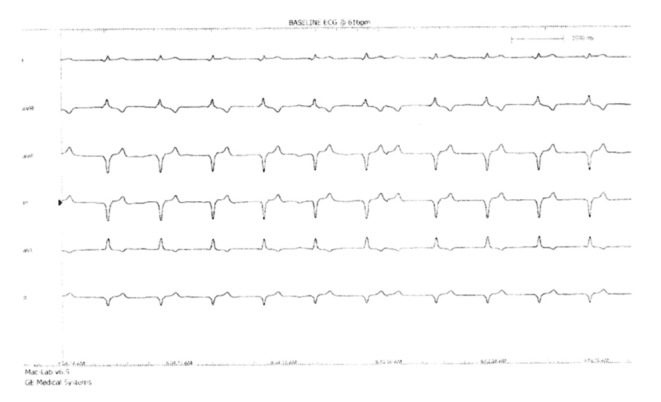
Paced QRS with sensed P wave at the end of the QRS, indicative of atrioventricular dyssynchrony.

On physical examination post pacemaker insertion, his blood pressure was 106/76 mmHg, with a heart rate (HR) of 76 bpm. The jugular venous pressure was elevated up to the angle of the jaw and cannon waves were present. His heart sounds were otherwise normal with no murmurs elicited, and there were no signs of heart failure. His chest was clear. The electrocardiogram (ECG) showed paced QRS with P wave at the end of the QRS complex, indicative of atrioventricular dyssynchrony (Fig. 1). These clinical findings, together with the ECG, raised the suspicion of pacemaker syndrome.

**Fig. 2.**
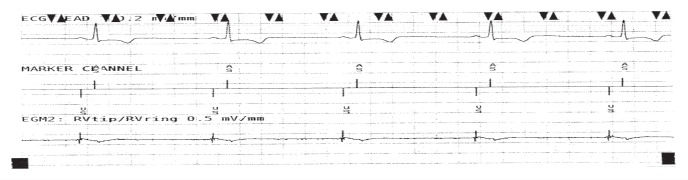
Electrogram showing typical right ventricular pacing, compatible with VVI mode despite AAIR-DDDR programming at the pacemaker. This shows ventricular pacing when in fact pacing is from the right atrial lead. There is `atrial sensing' from the ventricular lead.

Pacemaker interrogation showed that his pacemaker was programmed to AAIR–DDDR mode with a base rate of 70 bpm; the battery power was fine. The ECG showed typical right ventricular pacing compatible with VVIR mode despite AAIR– DDDR programming. Moreover, an atrial electrogram (EGM) showed ventricular pacing and a ventricular EGM showed sensed atrial depolarisation (Fig. 2). These findings were highly suggestive of atrial/ventricular lead switch at the pacemaker header. The underlying rhythm was sinus with an intrinsic rate of 45 bpm. Chest X-ray and fluoroscopy showed that the atrial and ventricular leads were situated in the correct positions in the respective chambers.

**Fig. 3.**
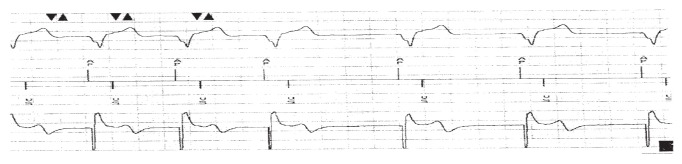
Twelve-lead ECG post lead repositioning showing proper atrial and ventricular pacing in AAIR-DDDR pacing mode.

The patient subsequently underwent a corrective procedure (lead repositioning) without temporary pacing cover. During the procedure, it was confirmed that the leads were switched, with the ventricular lead connected to the atrial port, and the atrial lead connected to the ventricular port. Both the atrial and ventricular leads were disconnected and tested, after which they were reconnected to the appropriate ports at the pacemaker header. Atrial pace and ventricular sense were achieved through the AAIR–DDDR pacing mode (Fig. 3).

It was noteworthy that prior to correction, the blood pressure was 108/62 mmHg, with a HR of 45 bpm. Post correction, the blood pressure immediately rose to 141/77 mmHg, with a HR of 72 bpm (AP–VS).

An echocardiogram done later the same day showed that the LVEF had dropped from 74 to 49% post pacemaker insertion. This high LVEF was thought to have been due to right ventricular-only pacing-induced left ventricular systolic dysfunction.

The differential diagnosis for pacemaker syndrome includes: acute coronary syndromes, hyperthyroidism, hypothyroidism, pacemaker failure, pacemaker-mediated tachycardia and cardiogenic pulmonary oedema, among others.5 In our case, the rise in systolic blood pressure of > 20 mmHg post correction of the leads confirmed the diagnosis of pacemaker syndrome.

The above differential diagnoses were ruled out as follows: the cardiac biomarkers were negative with no ST changes on the ECG, which ruled out an acute coronary syndrome. The patient was biochemically euthyroid with no clinical features of hyper- or hypothyroidism. The pacemaker was functional, ruling out the possibility of pacemaker failure. The resting heart rate was less than 100 bpm and this excluded the possibility of pacemaker syndrome in this case being due to pacemakermedicated tachycardia. The patient was clinically not in left ventricular failure, with clear lung fields on chest radiography and on auscultation, and therefore cardiogenic pulmonary oedema was an unlikely cause of his symptoms. Pulmonary embolism was also ruled out based on a negative D-dimer laboratory result.

## Discussion

Pacemaker syndrome is defined as intolerance to ventricularbased (VVIR) pacing due to loss of atrioventricular (AV) synchrony.^6^ It is an iatrogenic disorder that results from the haemodynamic sequelae of right ventricular-only pacing. Symptoms range from fatigability to syncope and occur during ventricular pacing. Postulated mechanisms include loss of AV synchrony, vasodepressor reflexes, and retrograde atrial activation. One of the ways to avoid pacemaker syndrome is maintenance of AV synchrony with a dual-chamber pacemaker with atrial tracking.

The overall incidence of pacemaker syndrome is unclear, with different studies reporting different results. In the Mode Selection Trial (MOST), pacemaker syndrome incidence was approximately 18%.^6^ According to Ausubel and Furman, the estimated incidence of pacemaker syndrome ranged from seven to 20%.^7^

The aetiology of pacemaker syndrome is poorly understood, but several risk factors are associated with its development:

Low intrinsic rate and high ventricular pacing rate, as noted in our patient, results in high percentage of ventricular pacing, therefore more AV dyssynchrony, and this may also explain the development of left ventricular systolic dysfunction.
Intact ventricular–atrial (VA) conduction poses a greater risk for the development of pacemaker syndrome.
Patients with non-compliant ventricles, such as in diastolic dysfunction, heart failure, hypertrophic cardiomyopathy, among others, are particularly sensitive to loss of atrial contribution to ventricular filling.^8^
Ventricular pacing leads to decreased cardiac output, with the resultant increase in left atrial pressure and left ventricular filling pressure.^5^

A major cause of AV dyssynchrony is VA conduction. Retrograde conduction leads to non-physiological timing of atrial contraction in relation to ventricular contraction. It should, however, be noted that many conditions other than VA conduction promote AV dyssynchrony.

Conventional non-physiological right ventricular pacing has deleterious effects on left ventricular systolic function.^2^ Yu et al. reported that conventional right ventricular apical pacing resulted in adverse left ventricular remodelling and therefore a reduction in LVEF in patients with normal systolic function.^3^

From the MOST and DAVID trials,^4^ it has become clear that a high amount of right ventricular apical pacing may be associated with a worse clinical outcome, including worsening left ventricular systolic function, new-onset congestive cardiac failure, as well as tachyarrhythmias, such as atrial fibrillation. Unfortunately, it remains unclear as to the exact amount of right ventricular apical pacing that negatively affects cardiac function.

## Conclusion

This case demonstrated that switching of atrial and ventricular leads at the pacemaker header resulted in pacemaker syndrome in a patient with a dual-chamber permanent pacemaker. The syndrome was due to incorrectly connected leads, resulting in ventricular paced atrial sensed (VP-AS), essentially producing the VVI pacing with retrograde conduction and loss of AV synchrony. This case also illustrates the possibility of right ventricular pacing-induced left ventricular dysfunction and highlights the need to maintain a high level of concentration during device implantation.
